# Second-trimester urine nephrin:creatinine ratio versus soluble fms-like tyrosine kinase-1:placental growth factor ratio for prediction of preeclampsia among asymptomatic women

**DOI:** 10.1038/srep37442

**Published:** 2016-11-22

**Authors:** Tianyue Zhai, Itsuko Furuta, Kinuko Nakagawa, Takashi Kojima, Takeshi Umazume, Satoshi Ishikawa, Takahiro Yamada, Mamoru Morikawa, Hisanori Minakami

**Affiliations:** 1 Department of Obstetrics, Hokkaido University Graduate School of Medicine, Sapporo, Hokkaido, Japan

## Abstract

This prospective observational study compare urine nephrin:creatinine ratio (NCR, ng/mg) with serum soluble fms-like tyrosine kinase-1:placental growth factor ratio (FPR, pg/pg) for preeclampsia (PE) prediction among unselected asymptomatic pregnant women in 2^nd^ trimester. NCR and FPR were determined in 254 paired urine/blood samples collected simultaneously from 254 women at median gestational week (GW) 24 (range, 22–27) without hypertension or significant proteinuria in pregnancy (SPIP). Fifteen (5.9%) developed SPIP and hypertension at GW 34.0 (26.0–38.6) and 35.3 (27.6–38.6), respectively, and were diagnosed with PE at GW 35.7 (27.6–38.6). The 90^th^ percentile level determined in 239 women normotensive throughout pregnancy gave NCR (139) sensitivity and positive predictive values (PPV) of 60% (9/15) and 27% (9/33), while those for serum FPR (4.85) were 40% (6/15) and 20% (6/30), respectively. Relative risks (95%CI) of later PE were 10.0 (3.82–26.4; 27% [9/33] vs. 2.7% [6/221]) and 4.98 (1.91–13.0; 20% [6/30] vs. 4.0% [9/224]) for NCR-positive and FPR-positive women, respectively. Cut-offs suggested by ROC gave NCR (86.6) sensitivity and PPV of 87% (13/15) and 17% (13/79), and FPR (8.8) values of 40% (6/15) and 40% (6/15), respectively. Thus, 2^nd^ trimester NCR was superior to FPR for PE prediction.

Nephrin is a podocyte-specific transmembrane protein that is predominantly localized at the glomerular slit diaphragm of podocytes^1^ and can detach from the glomerular podocytes, especially in patients with proteinuric diseases, such as a preeclampsia (PE), and is excreted in the urine^2^,^3^,^4^,^5^,^6^. The podocytes are glomerular epithelial cells located at the outermost layer of the glomerular basement membrane (GBM)^7^ and form the final barrier to protein loss^8^. Glomerular podocytes with reduced nephrin expression are likely to detach from the GBM, and podocyturia as a result from podocytes detachment from the GBM is associated with increased nephrinuria and proteinuria in women with PE^9^,^10^. These suggested that glomerular podocytes that had shed nephrin were impending detachment from the GBM and the shed nephrin became nephrinuria. Most studies dealing with nephrinuria in pregnancy acknowledge the possibility of nephrinuria as a biomarker predicting onset of PE^3^,^5^,^6^.

Proteinuria increases gradually with advancing gestation even in normotensive women^6^,^11^. Some patients with PE first develop significant proteinuria in pregnancy (SPIP) defined as urine protein:creatinine ratio (PCR, mg/mg) >0.27 and subsequently hypertension^12^,^13^. Isolated gestational proteinuria defined as SPIP in the absence of hypertension is a strong risk factor for PE^13^ and a 2^nd^ trimester cut-off level of PCR < 0.27 can differentiate between women with higher and lower risks of later developing PE^6^.

Circulating maternal serum levels of soluble fms-like tyrosine kinase 1 (sFlt-1) are increased and placental growth factor (PlGF) levels are decreased in women with PE^14^,^15^. As higher serum sFlt-1:PlGF ratio (FPR) is associated with higher risk of later PE^16^,^17^,^18^,^19^,^20^,^21^,^22^,^23^,^24^, FPR can be used to differentiate between women at higher and lower risk of later PE^16^,^17^,^18^,^19^,^20^,^21^,^22^,^23^,^24^.

Thus, all three variables, i.e., nephrinuria corrected by urine creatinine (NCR), PCR, and FPR, can be used as biomarkers of PE. To our knowledge, however, there have been no studies comparing NCR with FPR with regard to prediction of later development of PE. The present study was performed to compare NCR with FPR as well as PCR with regard to prediction of later PE among unselected asymptomatic women.

## Results

Of the 254 women included in this study, 15 (5.9%) later developed SPIP at gestational week (GW) 34.0 (26.0–38.6) and hypertension at GW 35.3 (27.6–38.6) and were diagnosed with PE at GW 35.7 (27.6–38.6), while the remaining 239 women developed neither SPIP nor hypertension (Table 1). In the 15 women with later PE, five first developed SPIP and subsequently developed hypertension, two developed hypertension first and later developed SPIP, and the remaining eight developed SPIP and hypertension simultaneously within 1 week. The GW at blood/urine sampling did not differ between women that did and did not develop PE later (24.7[22.0–27.4] vs. 24.4[22.7–25.4], respectively).

**Figure 1:**
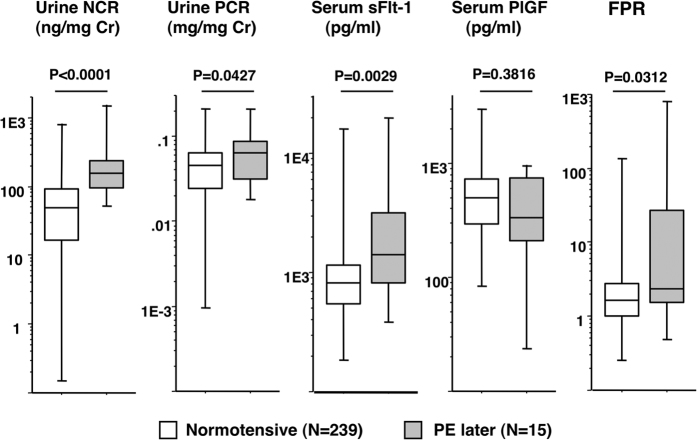
Second trimester NCR, PCR, sFlit-1, PlGF, and FPR levels in the 254 asymptomatic women. FPR, serum soluble (sFlit-1): placental growth factor (PlGF) ratio; urine NCR, spot-urine nephrin:creatinine ratio; urine PCR, spot-urine protein:creatinine ratio. Totals of 239 and 15 women with neither SPIP nor hypertension were examined at GW 25 (22–27) and 24 (23–25), respectively; the former 239 women remained normotensive throughout pregnancy and the latter 15 women later developed PE at GW 35.7 (27.6–38.6). Eleven and 22 urine specimens from PE and normotensive women, respectively, were used previously^6^, but nephrin concentrations in the 33 urines were measured again together with the remaining 221 samples for this study. All variable levels except PlGF differed significantly between the two groups. The median (range)/mean ± SD values in the 239 vs. 15 women were 49.6 (0.15–812)/69.6 ± 88.1 vs. 154 (52.2–1512)/261 ± 362 ng/mg Cr for NCR, 0.044 (0.001–0.215)/0.048 ± 0.031 vs. 0.062 (0.018–0.210)/0.073 ± 0.054 mg/mg Cr for PCR, 813 (192–1648)/1097 ± 1545 vs. 1414 (385–1969)/3440 ± 5027 pg/mL for sFlit-1, 497 (87–3034)/575 ± 397 vs. 336 (24–982)/474 ± 327 pg/mL for PlGF, and 1.65 (0.26–1334)/3.31 ± 10.3 vs. 2.32 (0.51–831)/64.6 ± 212 for FPR, respectively.

Although all 254 women were normotensive and not proteinuric at blood/urine sampling, all biomarkers except PlGF differed significantly between women that did and did not develop PE later (Fig. 1).

### Effect of number of days until nephrin assay after urine collection on NCR level

**Figure 2:**
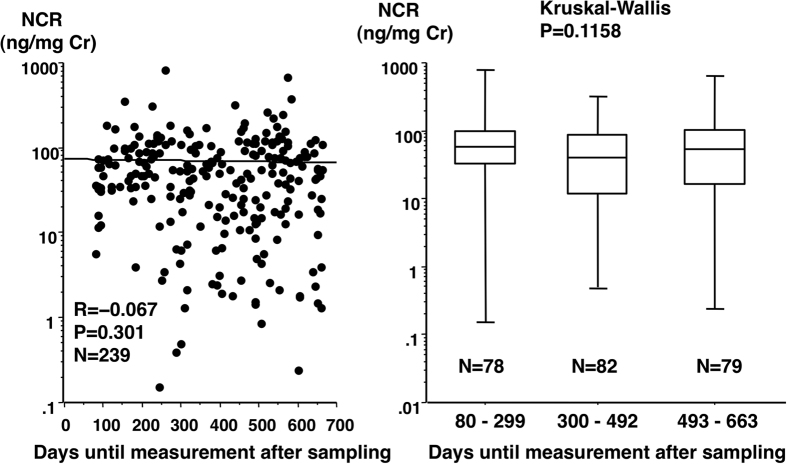
Effect of number of days until assay after specimen collection on urine NCR. Number of days until assay after urine collection varied from 80 to 662 in the 239 urines from 239 women who remained normotensive throughout pregnancy. There was no significant correlation between NCR levels and number of days after sampling until nephrin assay (Fig. 2, left). The median/mean NCR values were 57.2 (0.151–812)/79.9 ± 105, 40.0 (0.499–318)/55.5 ± 56.7, and 52.4 (0.239–671)/73.9 ± 96.0, for the 1^st^, 2^nd^, and 3^rd^ tertile groups divided by number of days until assay after sampling, respectively (Fig. 2, right). The GW at specimen sampling was 24.9 (23.4–27.4), 24.7 (22.0–26.7), and 24.9 (22.0–26.7) for the 1^st^, 2^nd^, and 3^rd^ tertile groups, respectively. Similar investigations on sFlit-1 and PlGF were performed (data not shown). There was no significant correlation of number of days after specimen sampling until assay with either sFlit-1 or PlGF level. The median/mean sFlit-1 values were 721 (250–16476)/1098 ± 1838, 813 (192–3268)/919 ± 622, and 882 (237–15986)/1277 ± 1846 pg/mL for the 1^st^, 2^nd^, and 3^rd^ tertile groups, respectively (*P* = 0.1776 with Kruskal-Wallis test). However, there was a significant difference in the median/mean values of PlGF with unknown reasons; 528 (123–3034)/656 ± 474, 348 (86.9–1405)/407 ± 264, and 638 (123–2244)/663 ± 373 pg/mL for the 1^st^, 2^nd^, and 3^rd^ tertile groups, respectively (*P* < 0.0001 with Kruskal-Wallis test).

We were concerned with degradation of urine nephrin detectable by our method with increasing number of days after specimen sampling. However, time intervals until assay of nephrin after sampling appeared not to affect the NCR level (Fig. 2).

### Ability of 2^nd^ trimester NCR, PCR, FPR, sFlt-1, and PlGF tests for differentiation of women with higher and lower risk of subsequent PE development

**Figure 3:**
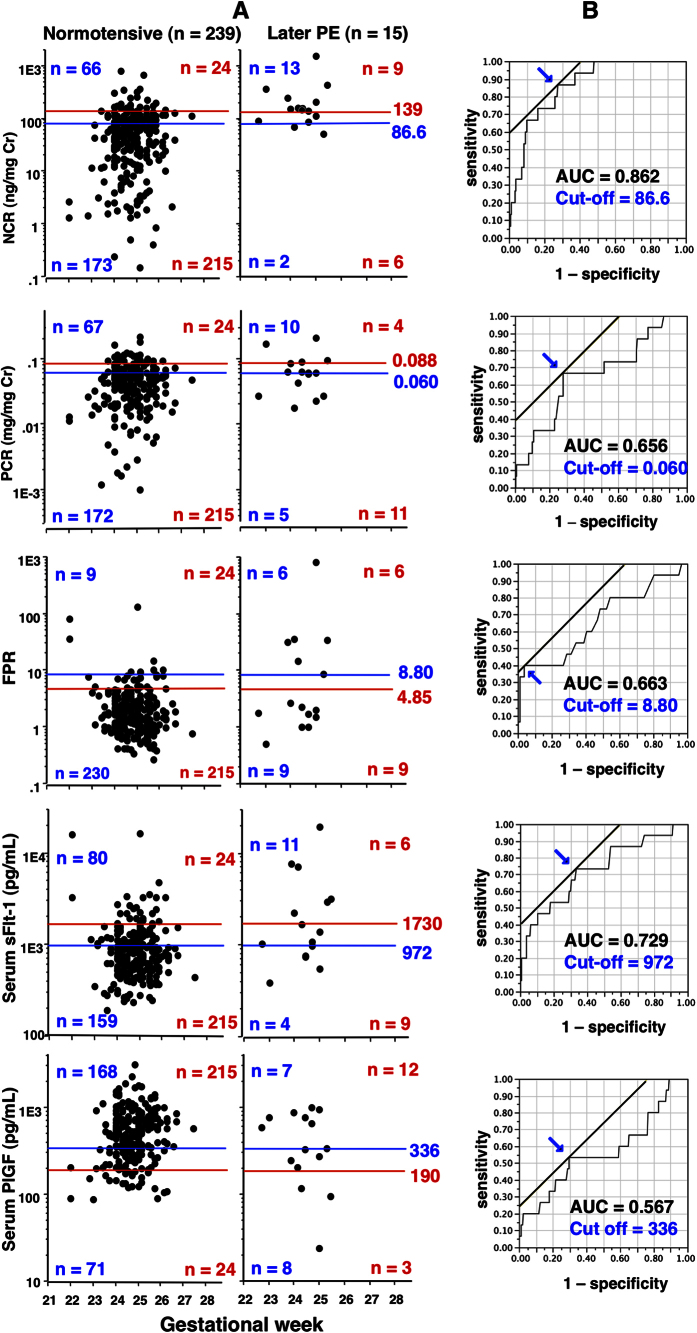
The 2^nd^ trimester NCR, PCR, FPR, sFlit-1, and PlGF tests as screening tools for detection of women at higher risk of PE. (**A**) Two horizontal lines (blue and red) indicate two cut-off levels: red is 90^th^/10^th^ percentile values obtained in the 239 women that remained normotensive throughout pregnancy, and blue is that suggested by the ROC shown on the right (**B**). Colored numerals indicate numbers of women with positive and negative test results divided by the corresponding colored lines (cut-off levels). (**B**) Cut-offs (indicated by arrows) determined based on ROC. See Table 2 for their screening characteristics.

All data on NCR, PCR, FPR, sFlt-1, and PlGF levels were plotted against GW at determination in women with and without later PE development (Fig. 3). We used two cut-off levels; i.e., the 90^th^ (for NCR, PCR, FPR, and sFlt-1) or 10^th^ percentile (for PlGF) levels in the 239 women without later PE development as shown by red lines in Fig. 3A; and the cut-off suggested by receiver operating characteristic curves (ROC) (Fig. 2B) shown as blue lines in Fig. 3A. The 90^th^ percentile NCR level was superior to the corresponding FPR level with regard to both sensitivity (60% vs. 40%, respectively) and PPV (27% vs. 20%, respectively) (Table 2). The relative risks (95% confidence interval) of later PE development were 10.0 (3.82–26.4; 27% [9/33] vs. 2.7% [6/221]) for the NCR test, 2.94 (1.00–8.60; 14% [4/28] vs. 4.9% [11/226]) for the PCR test, and 4.98 (1.91–13.0; 20% [6/30] vs. 4.0% [9/224]) for the FPR test in women with positive test results compared to those with negative test results in this setting. The area under the curve (AUC) of ROC was greater for NCR test than for FPR test (0.862 vs. 0.663, respectively) (Fig. 3B). The cut-off suggested by the ROC curve gave NCR test sensitivity and positive predictive values (PPV) of 87% (13/15) and 17% (13/79), respectively, while those for FPR were 40% (6/15) and 40% (6/15), respectively (Table 2).

### NCR, sFlt-1, PlGF, and FPR levels according to number of weeks before onset of SPIP and hypertension in 15 women that later developed PE

**Figure 4:**
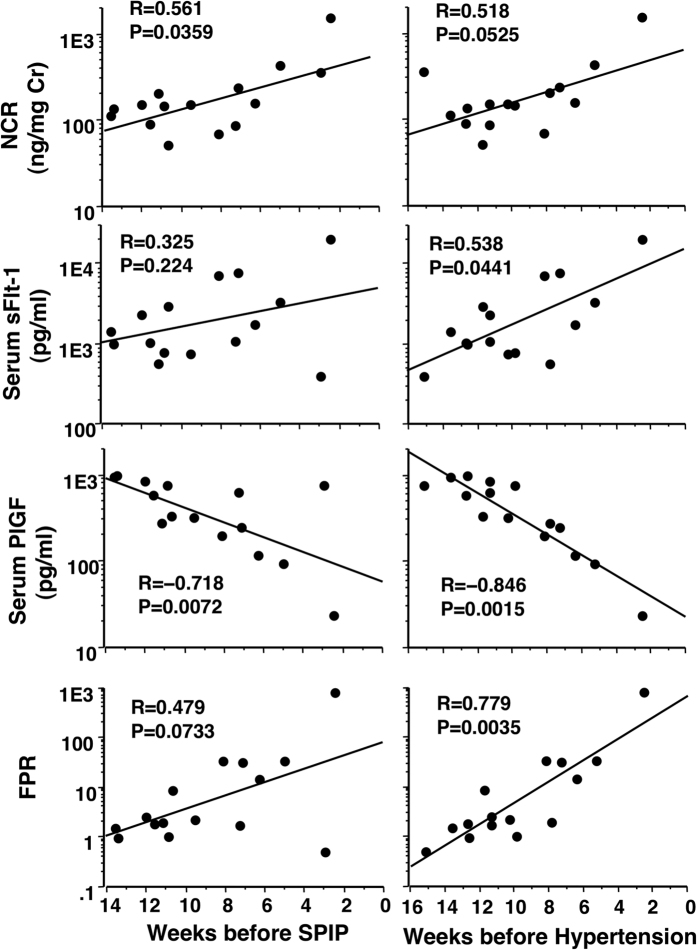
NCR, sFlt-1, PlGF, and FPR levels according to number of weeks before onset of SPIP and hypertension in 15 women with later development of PE. The NCR, but not sFlit-1 or FPR, increased significantly toward onset of SPIP in women with later development of PE. The sFlit-1 and FPR, but not NCR, increased significantly toward onset of hypertension. PlGF increased significantly toward both SPIP and hypertension.

NCR gradually increased significantly toward onset of SPIP, but not toward hypertension (Fig. 4). FPR increased significantly toward onset of hypertension, but not toward onset of SPIP. PlGF decreased significantly toward onset of both SPIP and hypertension (Fig. 4).

### Correlations of NCR with PCR, FPR, sFlt-1, and PlGF

**Figure 5:**
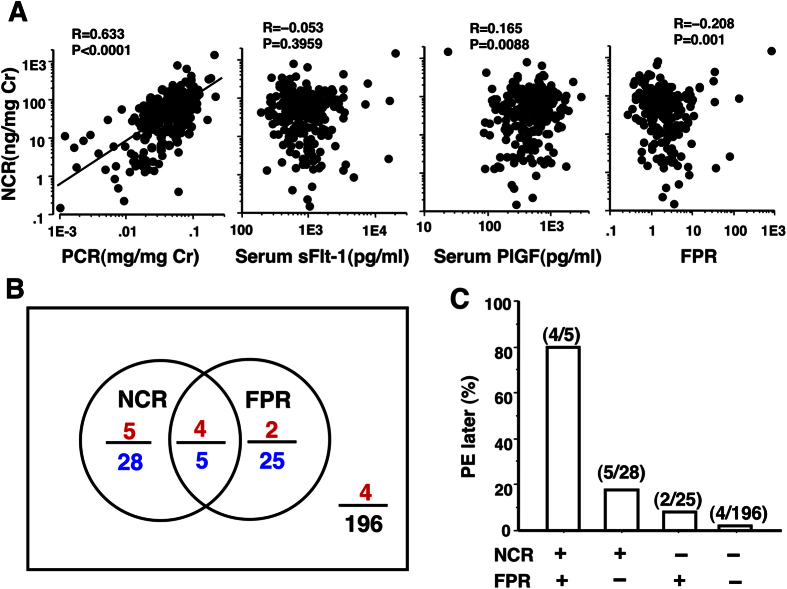
Correlations of NCR level with PCR, FPR, sFlit-1, and PlGF levels and predictability of NCR and FPR tests for subsequent PE development. (**A**) Correlations of NCR with other biomarkers; (**B**) Associations between NCR and FPR test results when the 90^th^ percentile values were used as cut-offs (see Fig. 3). Red, blue, and black numerals indicate numbers of women with later development of PE, positive tests results, and negative test result on both tests, respectively; (**C**), Risk of later PE development according to NCR and FPR test results: + and −, positive and negative test results, respectively.

Analyses of correlations of NCR with the other four variables, i.e., PCR, FPR, sFlt-1, and PlGF, indicated that the change in NCR level was almost independent of sFlit-1, PlGF, and FPR; *r* values were within the range of −2.49 to 2.49 (Fig. 5A). These suggested that combination of NCR with FPR increases positive predictive value (PPV) for prediction of later PE. When the 90^th^ percentile values were used as cut-offs of NCR and FPR tests, indeed as many as 80% (4/5) of women with positive results on both NCR and FPR tests later developed PE, while only 2.0% (4/197) of women with negative results on both NCR and FPR tests showed later development of PE (Fig. 5B,C) (Fig. 5).

## Discussion

To our knowledge, this is the first study to compare NCR with FPR with regard to ability to detect women at higher risk of PE among asymptomatic women.

The antiangiogenic factor, sFlit-1, and proangiogenic factor, PlGF, have been implicated in the pathogenesis of PE^25^. As FPR was consistently predictive of later PE in most studies^6^,^7^,^8^,^9^,^10^,^11^,^12^,^13^,^14^,^15^,^16^,^17^,^18^,^19^,^20^,^21^,^22^,^23^,^24^, FPR has been accepted as a diagnostic aid for PE in conjunction with other clinical findings^26^. This was also confirmed in the present study; women with FPR >90^th^ percentile value had RR of PE = 4.98 (1.91–13.0) compared to women with FPR <90^th^ percentile value.

The median values were 1414 vs. 813 pg/mL for sFlit-1 levels and 336 vs. 497 pg/mL for PlGF levels determined at around GW 24 in 15 vs. 239 women that did and did not later develop PE, respectively, in this study. These levels were similar to those determined in women with similar clinical conditions in a previous study by Levine *et al*.^16^, which used the identical assay kit to the present study. Therefore, absolute FPR levels around GW 25 in the two clinical conditions in this study were also similar to those reported by Levine *et al*.^15^. However, our absolute sFlit-1 value was somewhat lower and the PlGF value was similar to those proposed as normal values by Verlohhren *et al*.^26^ determined by different assay systems^26^; median sFlit-1 values were 1299 and 1355 pg/mL at GW 20–23 and GW 24–28, respectively, and corresponding PlGF values were 264 and 465 pg/mL, respectively, in the report by Verlohhren *et al*.^26^. Therefore, the assay systems used by Verlohhren *et al*.^26^ indicated higher normal FPR levels than our results: the median FPR levels were 4.92 and 3.06 at GW 20–23 and GW 24–28, respectively^26^, compared to 1.65 at GW 25 (22–27) in the present study.

The 2^nd^ trimester NCR around GW 24 appeared to be superior to simultaneous FPR with regard to the detection rate of women with later development of PE in our setting. Women with NCR > 90^th^ percentile value had RR of PE = 10.0 (3.82–26.4) compared to women with NCR < 90^th^ percentile value; the corresponding RR was 4.98 (1.91–13.0) for FPR in this study. In addition, AUC of ROC was greater for NCR than for FPR, and the NCR cut-off suggested by the ROC yielded high sensitivity of 87%. In our previous study using longitudinal urine samples from women that did and did not develop PE later^6^, the NCR did not change significantly in pregnant women that remained normotensive, while it increased with advancing gestation prior to the later development of PE, suggesting the possibility of using NCR as an urine biomarker of PE^6^. Therefore, NCR was considered worth comparing with the well-known biomarker, FPR, and the results indicated that NCR could be a clinically useful biomarker and may not be inferior to FPR. Screening characteristics of urinary nephrin for PE were examined in two previous studies with promising results^3^,^5^; sensitivity and specificity were 73% and 79%, respectively, in one study^3^, and 57% and 58%, respectively, in the other study for prediction of PE^5^. However, these findings should be confirmed in larger studies before clinical use, as performed for FPR^24^,^26^.

Unexpectedly, the 2^nd^ trimester NCR levels were independent of the 2^nd^ trimester sFlit-1, PlGR, and FPR (Fig. 5A). As exogenously administered sFlit-1 induces not only hypertension but also proteinuria associated with glomerular endotheliosis in pregnant rats^14^ and as increased NCR was associated with increased proteinuria (Fig. 4A), a significant correlation was expected between NCR and sFlit-1/FPR level. However, there were no significant correlations between 2^nd^ trimester NCR and 2^nd^ trimester sFlit-1/FPR level in this study. These unexpected results gave the combined use of NCR and FPR a high PPV of 80% for prediction of PE in this study (Fig. 5C). This was reasonable based on the findings that NCR was more predictive for SPIP than hypertension onset and that FPR was more predictive for hypertension than SPIP onset (Fig. 4). Thus, NCR and FPR had complementary roles to each other in detection of women with increased risk of PE.

## Study limitations

As our institution is a tertiary centre managing mainly women at higher risk, the frequency of PE (5.9%, 15/254) in this study was somewhat higher than the prevalence rate of 2.3% in the general Japanese population^27^. The number of women aged ≥35 years (44% [112/254)), with twin pregnancies (13% [32/254]), and with complications including DM/GDM and connective tissue diseases (approximately 20%) were greater in this study than in the general population. As all of these are risk factors for PE^27^,^28^,^29^, this observation explained the somewhat higher frequency of PE in this study, and therefore our study population did not represent the general population.

In conclusion, non-invasive urine test of NCR around GW 25 was compared with simultaneous serum FPR with regard to predictability of later development of PE. Results were promising and larger prospective studies are warranted regarding the ability of NCR for prediction of PE.

## Methods

This study was conducted in accordance with the principles of the Declaration of Helsinki and with the approval of the Institutional Review Board of Hokkaido University Hospital (013-3999, April 30, 2014), a tertiary teaching hospital managing mainly high-risk pregnant women.

### Participants

During a study period between from May 2014 to June 2016, 827 women received antenatal care and gave birth at Hokkaido University Hospital. Among them, 540 (65%) women gave written informed consent to participate in this study and provided at least one spot urine specimen with or without simultaneously collected blood specimen during pregnancy. From these 540 women, 97 women were first excluded from the present analyses: 15 women with known hypertension (pre-existing hypertension) at the establishment of the current pregnancy and 82 women that provided urine samples only collected during the 1^st^ or 3^rd^ trimester. Then, from the 443 women with availability of 2^nd^ trimester urine specimens but without chronic hypertension, the following 189 women were excluded: one woman that exhibited hypertension before reaching GW 20, three women that exhibited new onset of hypertension and or SPIP at the time of urine collection, and 185 women whose blood specimen collected simultaneously with urine collection was not available. Thus, the remaining 254 women fulfilling the following two criteria were finally enrolled in this study: (1) no known pre-existing hypertension, and (2) no hypertension or SPIP at the simultaneous urine and blood sampling during the 2^nd^ trimester. Thirty-three of the 254 women (13%) were also enrolled in our previous study^6^.

SPIP was defined as PCR (mg/mg) >0.27 (corresponding to 30 mg/mmol) in spot urine specimens. Hypertension was diagnosed in women with systolic blood pressure ≥140 mmHg and/or diastolic blood pressure ≥90 mmHg on at least two occasions recorded more than 12 hours apart. The GW at new onset of hypertension and SPIP were specified in each subject. PE was diagnosed in women that showed both hypertension and SPIP on and after GW 20.

### Biochemical procedures in the urine and blood specimens

All spot urine samples were coded and processed within 2 hours of collection. Urine samples were transferred into tubes and centrifuged at 700 × *g* for 5 minutes. Urinary supernatant was stored at –20 °C until measurement of protein, creatinine, and nephrin levels. Protein and creatinine concentrations were measured using a Protein Assay Rapid Kit Wako and Laboassay Creatinine (Wako Pure Chemical Industries, Osaka, Japan), respectively. Nephrin concentrations of all 254 urine samples were measured using a commercial ELISA kit (listed as #1035 in a catalog of Funakoshi Co. Ltd., Tokyo, Japan; human nephrin ELISA kit manufactured by Exocell, Philadelphia, Pennsylvania, USA) that was different from the kit used in our previous study^6^ (listed as #1019 in the catalog of Funakoshi Co. Ltd., Tokyo, Japan; nephrin ELISA kit manufactured by Exocell, Philadelphia, Pennsylvania, USA). Urine samples were diluted in 1:10 for nephrin assay. The range of the standard curve is 0.8–200 ng/mL according to the protocol of the human nephrin ELISA kit (listed as #1035 in the catalog of Funakoshi Co. Ltd.). The detection limit of urine nephrin concentration calculated as three times the standard deviation of reagent blank (measured by this kit) was 0.26 ng/mL. Similarly, the detection limit was 5 μg/mL for protein. We assumed that samples with undetectable levels contained 0.13 ng/mL nephrin and 2.5 μg/mL protein. Nephrin and protein concentrations in the urine were corrected by urine creatinine concentration and were expressed as nephrin:creatinine ratio (NCR, ng/mg) and protein:creatinine ratio (PCR, mg/mg). Serum samples collected at the time of urine collection were prepared according to the standard operating procedure and stored at –20 °C until measurement of sFlt-1 and PlGF using commercial ELISA kits (R&D Systems, Minneapolis, MN, USA). Serum samples were diluted in the range of 1:10–1:100 for sFlt-1 assay and 1:1–1:10 for PlGF assay.

### Statistical analyses

Data are presented as the median (range) and or mean ± SD. Statistical analyses were performed using the JMP10^©^ statistical software package (SAS, Cary, NC, USA). The Mann–Whitney U test was used to compare median or mean values between two groups. The Kruskal–Wallis test was used for comparison of medians or means of three groups. Receiver operating characteristic curves (ROC) were constructed for the biomarkers to assess their ability to differentiate women with PE onset later. The Spearman’s rank-order correlation was used to test associations between two variables. In all analyses, *P* < 0.05 was taken to indicate statistical significance. However, a significant finding regarding a linear correlation between two variables was defined as that meeting both *P* < 0.05 and correlation coefficient (*r*) > 0.25 or <–0.25.

## Additional Information

**How to cite this article**: Zhai, T. *et al*. Second-trimester urine nephrin:creatinine ratio versus soluble fms-like tyrosine kinase-1:placental growth factor ratio for prediction of preeclampsia among asymptomatic women. *Sci. Rep.* **6**, 37442; doi: 10.1038/srep37442 (2016).

**Publisher's note:** Springer Nature remains neutral with regard to jurisdictional claims in published maps and institutional affiliations.

## Figures and Tables

**Table 1:** Demographic characteristics of 254 women.

	Normotensive	Preeclampsia	*P*-value
No. of women	239	15	
Maternal age (years)	34 (20–53)	37 (27–43)	0.0978
≥35	102 (43%)	9 (60%)	0.1895
≥40	29 (12%)	7 (47%)	0.0002
Nulliparous	128 (53%)	15 (100%)	0.0004
Twin pregnancy	27 (11%)	5 (33%)	0.0126
Body height (m)	1.58 (1.38–1.77)	1.60 (1.53–1.66)	0.6685
Pre-pregnancy weight (kg)	52 (37–132)	52 (46–67)	0.8940
Body mass index (kg/m^2^)	20.4 (14.7–50.5)	20.5 (17.8–27.9)	0.9858
≥25	29 (12%)	2 (13%)	0.8905
Complications			
Diabetes mellitus (DM)	5 (2.1%)	1 (6.7%)	0.2578
Gestational DM	32 (13%)	2 (13%)	0.9951
Connective tissue diseases*	12 (5%)	3 (20%)	0.0170
GW at blood/urine sampling	24.7 (22.0–27.4)	24.4 (22.7–25.4)	0.0832
GW at onset			
Hypertension	NA	35.3 (27.6–38.6)	
SPIP	NA	34.0 (26.0–38.6)	
Preeclampsia	NA	35.7 (27.6–38.6)	
GW at delivery	38.4 (24.3–41.9)	36.9 (27.6–39.0)	<0.0001
<37	20 (8.4%)	8 (53%)	<0.0001
Infant birthweight (kg)	2.89 (0.48–4.23)	2.32 (0.70–2.86)	<0.0001

Data are presented as the median (range). GW, gestational week; NA, not applicable; SPIP, significant proteinuria in pregnancy defined as a protein-to-creatinine ratio (mg/mg) >0.27. *Connective tissue diseases included systemic lupus erythematosus, Sjogren’s syndrome, rheumatoid arthritis, and antiphospholipid syndrome.

**Table 2:** Screening characteristics of various parameters.

	Sensitivity	Specificity	PPV	NPV
*90*^*th*^*%tile values as cut-offs (10*^*th*^%tile for PlGF)*				
NCR [139 ng/mg]	60% (9/15)	90% (215/239)	27% (9/33)	97% (215/221)
FPR [4.85]	40% (6/15)	90% (215/239)	20% (6/30)	96% (215/224)
sFlit-1 [1730 pg/mL]	40% (6/15)	90% (215/239)	20% (6/30)	96% (215/224)
PlGF [190 pg/mL]	20% (3/15)	90% (215/239)	11% (3/27)	95% (215/227)
PCR [0.088]	27% (4/15)	90% (215/239)	14% (4/28)	95% (215/226)
*Cut-offs suggested by ROC curves*^†^				
NCR [86.6 ng/mg]	87% (13/15)	72% (173/239)	17% (13/79)	99% (173/175)
FPR [8.80]	40% (6/15)	96% (230/239)	40% (6/15)	96% (230/239)
sFlit-1 [972 pg/mL]	73% (11/15)	67% (159/239)	12% (11/91)	98% (159/163)
PlGF [336 pg/mL]	53% (8/15)	70% (168/239)	10% (8/79)	96% (168/175)
PCR [0.060]	67% (10/15)	72% (172/239)	13% (10/77)	97% (172/177)

PPV and NPV, positive and negative predictive values, respectively. Cut-off values are indicated in square brackets. *See Fig. 3A for the 90^th^ and 10^th^ percentile values obtained in the 239 women that remained normotensive throughout pregnancy; ^†^see Fig. 3B for ROC. In case of PlGF, positive test result was defined as at or below cut-off level.
